# Neural and Homeostatic Regulation of REM Sleep

**DOI:** 10.3389/fpsyg.2020.01662

**Published:** 2020-07-21

**Authors:** Sung-Ho Park, Franz Weber

**Affiliations:** Department of Neuroscience, Perelman School of Medicine, Chronobiology and Sleep Institute, University of Pennsylvania, Philadelphia, PA, United States

**Keywords:** sleep, REM sleep, neural circuits and behavior, REM sleep homeostasis, brain state

## Abstract

Rapid eye movement (REM) sleep is a distinct, homeostatically controlled brain state characterized by an activated electroencephalogram (EEG) in combination with paralysis of skeletal muscles and is associated with vivid dreaming. Understanding how REM sleep is controlled requires identification of the neural circuits underlying its initiation and maintenance, and delineation of the homeostatic processes regulating its expression on multiple timescales. Soon after its discovery in humans in 1953, the pons was demonstrated to be necessary and sufficient for the generation of REM sleep. But, especially within the last decade, researchers have identified further neural populations in the hypothalamus, midbrain, and medulla that regulate REM sleep by either promoting or suppressing this brain state. The discovery of these populations was greatly facilitated by the availability of novel technologies for the dissection of neural circuits. Recent quantitative models integrate findings about the activity and connectivity of key neurons and knowledge about homeostatic mechanisms to explain the dynamics underlying the recurrence of REM sleep. For the future, combining quantitative with experimental approaches to directly test model predictions and to refine existing models will greatly advance our understanding of the neural and homeostatic processes governing the regulation of REM sleep.

## Introduction

**FIGURE 1:**
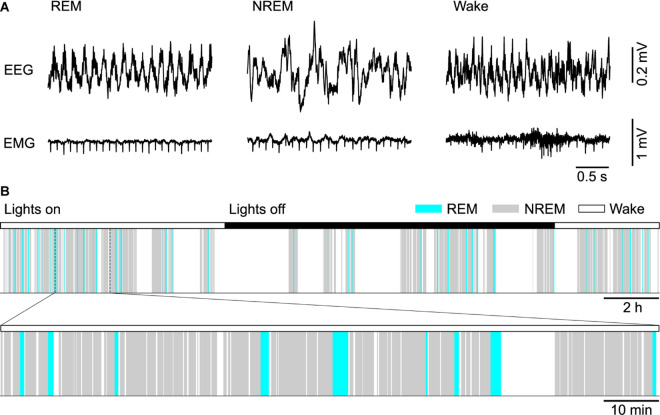
Sleep in mice. **(A)** Example electroencephalogram (EEG) and electromyogram (EMG) recordings from a mouse during REM sleep, NREM sleep and wakefulness (Wake). **(B)** Top, color-coded brain state (hypnogram) for a continuous 24 h recording from a mouse during dark and light cycles. Bottom, a 2 h segment of the hypnogram shown at an expanded scale.

In 1953, Aserinsky and Kleitman first reported the existence of rapid eye movement (REM) sleep in humans as a periodically recurring brain state marked by a low amplitude electroencephalogram (EEG) and rapid eye movements (Aserinsky and Kleitman, 1953). Soon afterward, Kleitman and Dement found that the EEG during REM sleep resembles that during alert waking and showed that REM sleep coincides with periods of vivid dreaming (Dement and Kleitman, 1957). Two years later, Jouvet discovered in cats that the activated EEG during REM sleep is associated with a complete paralysis of skeletal muscles, reflected in a flat electromyogram (EMG), and therefore coined the term paradoxical sleep (Jouvet and Michel, 1959). Besides these defining properties in EEG and EMG (Figure 1A), REM sleep is characterized by further striking neurophysiological and behavioral features, including high-amplitude theta oscillations in the hippocampus, muscle twitches, autonomic and respiratory activation, an elevated arousal threshold and bursts of large waves in the local field potential (LFP), called PGO-waves (or P-waves in rats), that originate in the pons and propagate to the lateral geniculate nucleus, occipital cortex, and other brain areas (Datta, 1997; Karashima et al., 2010).

In healthy subjects, REM sleep is always preceded by one or several non-REM (NREM) sleep periods. While asleep, the brain state recurrently alternates between these two major sleep states, constituting the ultradian NREM-REM or sleep cycle (Figure 1B). Until recently, the existence of two sleep states was thought to be unique to mammals and birds. Two recent studies in reptiles, however, discovered a REM sleep-like state marked by the occurrence of eye movements and unique cortical LFP features, in alternation with a NREM sleep-like state (Shein-Idelson et al., 2016; Libourel et al., 2018). Also in larval zebrafish, two sleep states sharing commonalities with mammalian NREM and REM sleep could be detected (Leung et al., 2019). Thus, the alternation between two distinct brain states throughout sleep might be a common characteristic of sleep in vertebrates. The highly regular sleep cycle in humans, lasting about 90 min, led Kleitman to interpret it as the manifestation of an underlying ultradian rhythm, termed the rest-activity cycle, that was thought to be implemented by an oscillator circuit that operates independently of sleep and possibly also modulates behaviors during wakefulness (Kleitman, 1963; Vyazovskiy and Tobler, 2012). McCarley and Hobson (1975) formulated the first physiologically based model of the sleep cycle, which implemented an oscillator circuit composed of two interacting neural populations. An alternative model posits that a homeostatic process drives the timing of REM sleep periods (Benington and Heller, 1994; Vivaldi et al., 1994). Homeostatic control of REM sleep timing implies that a propensity or pressure for REM sleep builds up in its absence and triggers a REM period if sufficient pressure has accumulated, followed by a discharge of pressure during REM sleep. Recent physiologically based models stress the importance of mutually inhibitory interactions between REM sleep-promoting (REM-on) and REM sleep-suppressing (REM-off) neurons in switching the brain state between NREM and REM sleep (Diniz Behn et al., 2013; Booth and Diniz Behn, 2014; Dunmyre et al., 2014; Weber, 2017; Héricé et al., 2019).

While we have learnt a lot about the identity of circuits involved in controlling REM sleep, their connectivity and activity during sleep, we still lack a clear understanding about the neural or homeostatic mechanisms that ultimately determine when a REM sleep period occurs. Consequently, the mechanisms underlying the mammalian sleep cycle also remain elusive. More generally, our knowledge about how the brain generates ultradian rhythms on a minute–to–hour time scale is limited, a fundamental gap in our understanding of brain rhythms.

Over the past decade, the availability of new technologies, including optogenetics, chemogenetics, calcium imaging, gene profiling and viral tracing, endowed us with the capability to manipulate, record and map the connectivity of genetically identified neurons at an unprecedented level of detail (Weber and Dan, 2016; Peever and Fuller, 2017; Shiromani and Peever, 2017), greatly advancing the investigation of the neural mechanisms regulating mammalian sleep. Here we review the key neural populations thought to control REM sleep, with a special focus on recent studies, and summarize findings about homeostatic processes regulating REM sleep. Finally, we focus on a recently proposed model for REM sleep regulation that integrates current knowledge about the neural and homeostatic mechanisms governing the timing of REM sleep and discuss recent experimental results supporting its predictions.

## Neural Control of REM Sleep

Research over the past decades has identified, with increasing precision, key areas and neural populations involved in the control of REM sleep (Saper et al., 2010; Luppi et al., 2012; Peever and Fuller, 2017; Scammell et al., 2017). The core circuitry generating REM sleep is localized in the brainstem, but populations of neurons powerfully regulating REM sleep by either promoting or suppressing its occurrence have been found throughout the medulla, pons, midbrain, and hypothalamus.

### Excitatory Circuits Controlling REM Sleep

Seminal transection studies by Jouvet identified the dorsolateral pons as a crucial region for REM sleep generation (Jouvet, 1962). He could further demonstrate that lesions in this area abolish the muscle paralysis during REM sleep (Jouvet and Delorme, 1965), such that lesioned animals seemed to act out their dreams. Subsequent work further refined the location of this area (Mouret et al., 1967; Henley and Morrison, 1974; Hendricks et al., 1982; Sanford et al., 2001), known as the subcoeruleus area in cats or also the sublaterodorsal nucleus (SLD) in rodents. Chemogenetic inhibition of SLD neurons in mice has been recently shown to increase the muscle tone during REM sleep, while chemogenetic activation triggered cataplexy, a sudden weakening of the skeletal muscle tone during wakefulness (Torontali et al., 2019). Rodent studies established that glutamatergic neurons within the SLD trigger muscle paralysis during REM sleep (Clément et al., 2011; Krenzer et al., 2011; Valencia Garcia et al., 2017). Selective disruption of glutamatergic transmission of SLD neurons through conditional knockout of *Vglut2* (the gene encoding the vesicular glutamate transporter 2) or knockdown using RNA interference increased the muscle tone and triggered motor activity during REM sleep (Krenzer et al., 2011; Valencia Garcia et al., 2017), suggesting that glutamatergic neurons in the SLD induce muscle atonia, either through direct projections to the spinal cord (Lu et al., 2006) or through activation of inhibitory, glycinergic neurons in the ventromedial medulla (Valencia Garcia et al., 2018). Consistent with a role in generating muscle atonia, glutamatergic SLD neurons have been shown to express elevated levels of the immediate early gene c-Fos following deprivation-induced REM sleep rebound (Clément et al., 2011). Juxtacellular *in vivo* recordings in rats and calcium imaging of VGLUT2 neurons expressing the genetically encoded calcium indicator GCaMP6 using microendoscopes in freely moving mice confirmed the existence of REM sleep-active glutamatergic neurons in the dorsolateral pons including the SLD (Boucetta et al., 2014; Cox et al., 2016). Disrupting glutamatergic signaling in the SLD not only abolished muscle atonia, but also caused a fragmentation of REM sleep and reduced its amount (Krenzer et al., 2011; Valencia Garcia et al., 2017), suggesting that glutamatergic SLD neurons are also important for regulating REM sleep itself. Consistent with this, a recent study found that chemogenetic activation of SLD VGLUT2 neurons increases REM sleep (Erickson et al., 2019).

Cholinergic neurons in the dorsolateral pons have also been extensively studied for their role in REM sleep regulation (Brown et al., 2012; Peever and Fuller, 2017). Juxtacellular recordings and calcium imaging demonstrated that cholinergic neurons in the pedunculopontine tegmentum (PPT) and laterodorsal tegmentum (LDT) are wake- and REM sleep-active (Boucetta et al., 2014; Cox et al., 2016; Tsunematsu et al., 2020). Early pharmacological experiments showed a powerful effect of cholinergic agonists on REM sleep induction (George et al., 1964; Baghdoyan et al., 1984; Vanni-Mercier et al., 1989; Bourgin et al., 1995; Kubin, 2001). In addition, optogenetic activation of cholinergic neurons in the LDT/PPT increased the probability of NREM to REM transitions, but not the duration of REM sleep periods (Van Dort et al., 2015). Chemogenetic activation of these neurons, however, was found to promote light NREM sleep instead (Kroeger et al., 2017) and chemogenetic inhibition had no effect on the sleep architecture, consistent with lesion and pharmacological studies antagonizing muscarinic acetylcholine receptors in the pons (Lu et al., 2006; Grace et al., 2014). However, knockout of the muscarinic acetylcholine receptor 3 reduced REM sleep (Goutagny et al., 2005) and a recent study showed that double knockout of muscarinic receptors 1 and 3 completely abolishes REM sleep (Niwa et al., 2018), suggesting a necessary role of cholinergic signaling in REM sleep control. While signs of REM sleep were completely absent in the cortical EEG, other subcortical signatures of REM sleep may still be present. Hence, while cholinergic signaling within the pons might not be required for REM sleep, cholinergic receptors in other areas may be necessary for the expression of REM sleep in the forebrain.

### Suppression of REM Sleep by REM-Off Neurons

Disinhibition of the SLD by GABA_A_ receptor antagonists induces a long-lasting REM sleep-like state with EEG desynchronization and muscle atonia (Boissard et al., 2002), indicating that GABAergic neurons presynaptic to the SLD may powerfully suppress REM sleep. Anatomical tracing revealed a strong projection from the vlPAG and the neighboring deep mesencephalic reticular nucleus (DpMe) to the dorsolateral pons (Boissard et al., 2003; Lu et al., 2006; Hayashi et al., 2015) and rabies-virus mediated monosynaptic, retrograde tracing confirmed that GABAergic neurons in the vlPAG directly innervate SLD glutamatergic neurons (Weber et al., 2018). Inhibition of the vlPAG/DpMe through muscimol injection and lesioning of neurons resulted in a strong increase in REM sleep in mice, rats, cats, and guinea pigs (Petitjean et al., 1975; Sastre et al., 1996; Crochet et al., 2006; Lu et al., 2006; Vanini et al., 2007; Kaur et al., 2009; Sapin et al., 2009), whereas disinhibition of this area with the GABA_A_ receptor antagonist bicuculline decreased REM sleep (Vanini et al., 2007), suggesting a prominent role of the vlPAG in suppressing REM sleep. Recent studies confirmed that specifically suppressing the activity of GABAergic neurons in the vlPAG/DpMe using optogenetic or chemogenetic approaches or cell-type specific lesioning increased REM sleep (Hayashi et al., 2015; Weber et al., 2015, 2018). Conversely, opto- or chemogenetic activation of vlPAG GABAergic neurons strongly suppressed REM sleep, while promoting NREM sleep. Closer analysis of the optogenetic effect demonstrated that activation of vlPAG GABAergic neurons consolidated NREM sleep by suppressing transitions from NREM to REM sleep or wakefulness and impaired the maintenance of REM sleep (Weber et al., 2018). These findings thus argue that vlPAG GABAergic neurons antagonize REM sleep by both preventing its initiation and maintenance. Cell-type specific electrophysiological recordings using optogenetic tagging and calcium imaging with microendoscopes demonstrated that most vlPAG GABAergic neurons were strongly suppressed during REM sleep. Their activity gradually decayed during NREM sleep, was lowest at the onset of REM sleep and was abruptly activated at the termination of REM sleep (Weber et al., 2018). Such a temporal profile further lends support to their role as REM-off neurons and suggests that inhibition of vlPAG GABAergic neurons during natural sleep enables transitions into REM sleep.

Before the discovery of REM-off neurons in the vlPAG, monoaminergic neurons in the nearby dorsal raphe (DR) and locus coeruleus (LC) had been proposed to suppress REM sleep (McCarley and Hobson, 1975; McCarley and Massaquoi, 1986). In support of the idea that serotonin antagonizes REM sleep, perfusion of serotonin in the LDT reduced REM sleep in rats (Horner et al., 1997). Electrophysiological *in vivo* recordings showed that putative serotonergic neurons in the DR (McGinty and Harper, 1976; Trulson and Jacobs, 1979; Lydic et al., 1984; Sakai and Crochet, 2001) and other raphe nuclei (Trulson and Trulson, 1982; Sakai, 2018) have lowest activity during REM sleep. A recent study confirmed this REM-off activity profile by recording the population calcium activity of DR serotonergic neurons expressing GCaMP6 using fiber photometry (Oikonomou et al., 2019). Similar to that of vlPAG GABAergic neurons, the activity of DR serotonergic neurons gradually decreases during NREM sleep bouts, is lowest before entering REM sleep and abruptly rises at the end of REM sleep (Trulson and Jacobs, 1979; Oikonomou et al., 2019). Moreover, optogenetic activation using a regular tonic laser protocol (stimulation at 3 Hz) suppressed REM sleep, while enhancing NREM sleep, similar to the effects observed for activating vlPAG GABAergic neurons (Oikonomou et al., 2019). In contrast, presenting the laser pulses in bursts to mimic burst firing increased the time spent in wakefulness, indicating that the precise activity pattern of DR neurons determines their effect on the brain state. Ablation of serotonergic neurons, either in the dorsal and medial raphe or the whole brain also decreased the amount of REM sleep, by reducing the number of NREM to REM transitions, while increasing NREM to wake transitions (Iwasaki et al., 2018; Oikonomou et al., 2019). Hence, while activation of these neurons suppresses REM sleep and maintains NREM sleep, their presence seems to be paradoxically required for the normal expression of REM sleep.

Another population of monoaminergic neurons originally proposed to oppose REM sleep as REM-off neurons are the noradrenergic neurons in the LC. *In vivo* recordings revealed a state-dependent activity profile similar to that of DR serotonergic or vlPAG GABAergic neurons: A slow decay of firing throughout NREM sleep, followed by lowest activity during REM sleep and an abrupt activity increase at the end (Aston-Jones and Bloom, 1981). Pharmacological experiments provided evidence that norepinephrine suppresses REM sleep. Injection of norepinephrine into the peri-LC alpha within the subcoeruleus in cats reduced REM sleep, similar to the effects observed for agonists for both alpha1 and alpha2 adrenergic receptors (Cirelli et al., 1992; Crochet and Sakai, 1999). However, intraventricular injection of norepinephrine or administration into the forebrain promoted wakefulness (Segal and Mandell, 1970; Flicker and Geyer, 1982). Similarly, opto- or chemogenetic activation of noradrenergic LC neurons strongly promoted wakefulness (Carter et al., 2010; Gompf et al., 2015), consistent with the results observed for pharmacological activation of LC neurons in anesthetized rats (Berridge and Foote, 1991). Using CRISPR/Cas9 technology to disrupt the gene for dopamine beta hydroxylase (*dbh*, an enzyme necessary for norepinephrine synthesis) delayed the arousal following optogenetic stimulation during NREM sleep, suggesting that norepinephrine is important for the awakening effect observed for LC activation (Yamaguchi et al., 2018). The observed suppression of REM sleep by norepinephrine may thus be indirect through its wake-promoting effect and direct through inhibition of REM-on neurons in the peri-LC alpha, as shown by combining microdialysis with *in vivo* recordings in cats (Sakai and Koyama, 1996). Although LC neurons and norepinephrine suppress REM sleep, lesioning or optogenetic inhibition did not increase its amount (Lu et al., 2006; Carter et al., 2010). Similarly, disrupting norepinephrine signaling through knockout or CRISPR/Cas9-mediated knockdown of *dbh* had no effects on REM sleep (Hunsley and Palmiter, 2003; Yamaguchi et al., 2018).

A further brainstem area with NREM-promoting neurons that antagonize REM sleep is the parafacial zone (PZ) in the dorsorostral medulla. Chemogenetic activation of PZ GABAergic neurons strongly promoted NREM sleep and enhanced EEG slow wave activity (SWA), while completely suppressing REM sleep for hours (Anaclet et al., 2014, 2018). Conditional knockout of *Vgat* (vesicular GABA/glycine transporter) in PZ neurons strongly increased wakefulness, possibly explaining the resulting reduction in REM sleep (Anaclet et al., 2012). Finally, opto- and chemogenetic activation of GABAergic neurons in the ventromedial medulla has also been demonstrated to suppress REM sleep, while promoting NREM sleep (Chen et al., 2017; Zhong et al., 2019). However, chemo- or optogenetic inhibition of GABAergic GAD2 neurons did not increase REM sleep (Zhong et al., 2019). The circuit mechanisms mediating the suppression of REM sleep by ventromedial medulla neurons are still unclear. One possibility is that these REM-off neurons locally interact with nearby REM-on neurons in the ventral medulla (Weber et al., 2015).

Besides neurons regulating REM and NREM sleep, the ventral medulla also comprises a subpopulation of neurons triggering muscle atonia (Magoun and Rhines, 1946; Schenkel and Siegel, 1989). Glycinergic neurons in the ventromedial medulla are activated during REM sleep, project to the spinal cord (Holstege and Bongers, 1991; Sapin et al., 2009; Valencia Garcia et al., 2018) and hyperpolarize somatic motor neurons during REM sleep (Chase et al., 1989). Experiments combining c-Fos staining with retrograde tracing suggest that neurons in the nearby lateral paragigantocellular nucleus (LPGi) suppress motor neurons in the facial nucleus during REM sleep (Sirieix et al., 2012). The different inhibitory subpopulations in the ventral medulla differ in their postsynaptic projection targets: spinally projecting neurons suppress somatic motor neurons (Holstege and Bongers, 1991; Valencia Garcia et al., 2018), the NREM sleep-promoting neurons innervate wake-promoting neurons (Zhong et al., 2019), and vlPAG-projecting neurons promote REM sleep (Weber et al., 2015). Future work is needed to identify more refined molecular markers defining these different subpopulations of medulla neurons.

The brainstem also contains excitatory REM-off neurons, which express the neuropeptide neurotensin (NTS). Chemogenetic activation of NTS neurons in the sublaterodorsal tegmentum (subLDT) promoted NREM sleep, while reducing the amount of REM sleep (Kashiwagi et al., 2020). The NTS neurons are glutamatergic and overlap with neurons that are derived from proneural hindbrain cells expressing *Atoh1.* These neurons project to the vlPAG/DpMe and may thus suppress REM sleep through excitation of REM-off neurons (Hayashi et al., 2015). Interestingly, opto- or chemogenetic activation of NTS-neurons in other brainstem areas including vlPAG, lateral PAG, DpMe, or medial vestibular nucleus had similar effects, indicating that they form a wide-spread network of NREM-promoting brainstem neurons (Kashiwagi et al., 2020). Optrode recordings and calcium imaging using microendoscopy showed that the majority of the vlPAG NTS neurons are most active during NREM sleep (Zhong et al., 2019). Opto- or chemogenetic inhibition of NTS neurons in the vlPAG suppressed NREM sleep but did not cause an increase in REM sleep (Zhong et al., 2019). Similarly, disrupting synaptic signaling of the subLDT NTS neurons through expression of tetanus toxin light chain decreased NREM sleep. REM sleep was increased during the light and reduced during the dark phase, such that its overall amount was unchanged (Kashiwagi et al., 2020).

Altogether, the brainstem comprises several neural populations that promote NREM sleep while suppressing REM. However, among those, only inhibition or ablation of vlPAG GABAergic neurons results in an increase in REM sleep, indicating that these neurons play both a sufficient and necessary role in gating REM sleep and thus constitute a key node in the REM sleep circuitry.

### Inhibitory REM-on Populations

**FIGURE 2:**
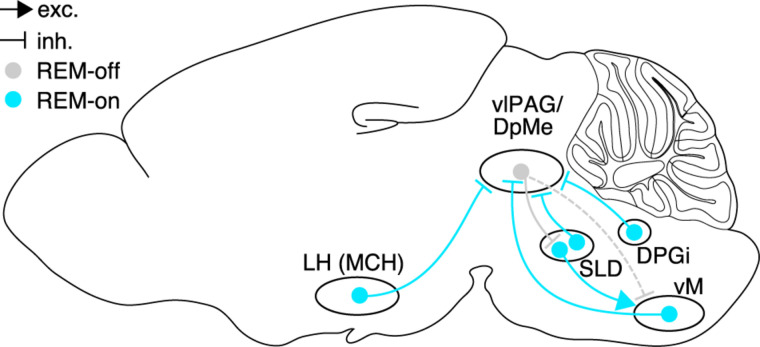
Brain circuits controlling REM sleep. Neural populations in hypothalamus, pons, and medulla promoting REM sleep (REM-on, cyan) or suppressing REM sleep (REM-off, gray), and their synaptic interactions. Glutamatergic neurons in the SLD are strongly inhibited by GABAergic REM-off neurons in the vlPAG/DpMe, which in turn are inhibited by GABAergic REM-on populations in hypothalamus (MCH neurons), pons (SLD), and dorsal (DPGi) and ventral medulla. Rabies virus-mediated retrograde tracing confirmed that vM GABAergic neurons innervate vlPAG GABAergic neurons, and that vlPAG GABAergic neurons synapse onto SLD glutamatergic neurons. Dashed lines indicate the existence of axonal projections whose functional role, however, has not been tested yet. Sagittal brain scheme adapted from Allen Mouse Brain Atlas (© 2015 Allen Institute for Brain Science. Allen Brain Atlas API. Available from: http://brain-map.org/api/index.html).

The strong suppression of REM sleep by vlPAG GABAergic neurons suggests that inhibition of the vlPAG by presynaptic neurons may directly contribute to the induction of REM sleep. Research in the last years has indeed identified several brain areas containing inhibitory REM-on neurons, most of which share the vlPAG as a common postsynaptic target and thus likely trigger REM sleep through inhibition of REM-off neurons in the vlPAG (Figure 2).

The lateral hypothalamus (LH) comprises a large number of GABAergic neurons that express c-Fos following deprivation induced REM sleep rebound (Sapin et al., 2010) and pharmacological inhibition of the LH reduced the amount of REM sleep, suggesting a prominent role of this area in REM sleep regulation (Clément et al., 2012). A subpopulation of the GABAergic, c-Fos-labeled neurons expresses the neuropeptide melanin-concentrating hormone (MCH) (Sapin et al., 2010; Clément et al., 2012) and intracerebroventricular administration of MCH increased the amount of REM and NREM sleep (Verret et al., 2003). *In vivo* calcium imaging using microendoscopy confirmed that MCH neurons are consistently most active during REM sleep (Blanco-Centurion et al., 2019). Contrary to these findings, another study using calcium imaging reported the existence of a second subpopulation of MCH neurons only activated during wakefulness (Izawa et al., 2019). Gene expression studies suggested the existence of two MCH subpopulations, one of which is defined by the expression of the neurokinin 3 receptor (NK3) and the cocaine- and amphetamine-regulated transcript (CART) peptide (Mickelsen et al., 2019). Interestingly, only neurons belonging to this subpopulation project to the periaqueductal gray (Cvetkovic et al., 2004), but neurons with elevated levels of c-Fos after a REM sleep rebound were found in both subpopulations (Hanriot et al., 2007).

Optogenetic activation of MCH neurons increased the number of NREM to REM sleep transitions during laser stimulation (Tsunematsu et al., 2014) and extended the duration of REM sleep episodes (Jego et al., 2013), indicating that these neurons promote both the initiation and maintenance of REM sleep. Chronic optogenetic activation of MCH neurons over 24 h enhanced NREM as well as REM sleep (Konadhode et al., 2013; Blanco-Centurion et al., 2016). Chemogenetic activation of MCH neurons instead only increased the percentage of REM sleep without affecting that of NREM sleep (Vetrivelan et al., 2016; Kroeger et al., 2019). A recent study reported a shortened duration of NREM sleep bouts, indicating that MCH neuron activation facilitates transitions from NREM to REM sleep (Varin et al., 2018). In contrast, chemogenetic inhibition of MCH neurons reduced the amount of REM sleep and instead increased the duration of NREM sleep bouts. Consistent with this, optogenetic inhibition of the axonal terminals of MCH neurons in the vlPAG during NREM sleep reduced the likelihood of transitions from NREM to REM sleep (Kroeger et al., 2019), further supporting that these neurons facilitate transitions into REM sleep. Unexpectedly, partial ablation of MCH neurons using cell-type-specific expression of diphtheria toxin A increased REM sleep during the light period and severely fragmented NREM sleep (Varin et al., 2016). The discrepancy with chemogenetic inhibition might arise from compensatory mechanisms in response to the chronic loss of MCH neurons as opposed to the acute chemogenetic manipulation. Interestingly, optogenetic inhibition of MCH neurons and knockout of MCH receptors block an increase in REM sleep typically observed for a warming in ambient temperatures (Komagata et al., 2019), suggesting that the MCH system adjusts the amount of REM sleep to the needs arising from the external environment.

As a mechanism by which MCH neurons enhance REM sleep, recent studies suggest that they inhibit the vlPAG, resulting in disinhibition of the SLD (Clément et al., 2012; Luppi et al., 2013; Kroeger et al., 2019). Anatomical tracing showed that a large proportion of neurons in the LH projecting to the vlPAG/DpMe express MCH (Clément et al., 2012). A recent study combined chemogenetic activation of MCH neurons with optogenetic suppression of MCH axon terminals in the vlPAG (Kroeger et al., 2019). Inhibition of MCH axon terminals during NREM sleep reduced the number of NREM to REM sleep transitions to comparable levels irrespective of whether MCH neurons were chemogenetically excited or not, suggesting that their REM sleep-promoting effect is indeed mediated via their projections to the vlPAG/DpMe. Inhibition of LC neurons has been proposed as an alternative mechanism by which the MCH system promotes REM sleep, as microinjection of the neuropeptide MCH into the LC increases REM sleep (Monti et al., 2015).

Inhibitory neurons involved in REM sleep control have also been found in the preoptic area of the hypothalamus. Lesions in an area dorsal and medial to the cluster of sleep-active neurons in the ventrolateral preoptic area (vlPO) reduced the amount of REM sleep (Lu et al., 2000) and the number of c-Fos labeled cells in this area was correlated with the amount of REM sleep (Lu et al., 2002). The axons of neurons in this region project to the DR, LC, and vlPAG (Lu et al., 2002; Hsieh et al., 2011) and may thus also promote REM sleep through inhibition of REM-off neurons in these areas. In the dorsomedial hypothalamic nucleus (DMH), a subpopulation of galanin-expressing GABAergic neurons that project to the raphe pallidus, a brainstem area containing serotonergic neurons with lowest activity during REM sleep (Trulson and Trulson, 1982), is preferentially active during REM sleep, and optogenetic activation of these projection neurons increases REM sleep (Chen et al., 2018). A separate GABAergic population in the zona incerta (ZI), which expresses the LIM homeodomain factor Lhx6, promotes both NREM and REM sleep as shown by chemogenetic activation and inhibition experiments (Liu et al., 2017) and express elevated levels of c-Fos during REM sleep hypersomnia (Lee et al., 2020). Lhx6 neurons inhibit nearby wake-promoting orexin/hypocretin and GABAergic neurons in the LH and their axons project to wake-promoting populations in the midbrain. Their axonal projections to areas with REM-off neurons including the dorsal raphe and vlPAG may underlie their REM sleep-promoting effect.

Inhibitory REM-on neurons have also been identified in the medulla. Optogenetic activation of GAD2 neurons in ventral medulla and their rostral projections to the vlPAG triggered a marked increase in NREM to REM transitions (Weber et al., 2015). These GAD2 neurons were targeted more laterally within the LPGi compared with studies investigating the role of the ventromedial medulla neurons in regulating NREM sleep or muscle atonia (Chen et al., 2017; Valencia Garcia et al., 2018; Zhong et al., 2019). Closed-loop optogenetic activation or inhibition of ventral medulla REM-on neurons was found to respectively prolong or shorten the duration of single REM sleep episodes, indicating that these neurons are also important for maintaining REM sleep. Optrode recordings from channelrhodopsin 2 (ChR2)-tagged neurons further showed that GAD2 neurons in the ventral medulla are highly active during REM sleep. Their firing rates increased gradually over 30 s before the onset of REM sleep, were sustained at a high level throughout REM sleep, and abruptly decayed as REM sleep ended. Trans-synaptic retrograde tracing with a modified rabies virus demonstrated that inhibitory ventral medulla neurons innervate GABAergic neurons in the vlPAG and optogenetic activation of the axonal projections to the vlPAG reproduced the effects on initiation and maintenance, indicating that vM neurons enhance REM sleep, at least in part, by inhibition of vlPAG REM-off neurons.

The dorsomedial medulla has also been implicated in regulating REM sleep. Electrophysiological *in vivo* recordings in head-fixed rats and mice revealed that this area contains neurons that are strongly activated during REM sleep (Goutagny et al., 2008; Sakai, 2018). GABAergic neurons in the dorsal paragigantocellular reticular nucleus (DPGi) within the dorsomedial medulla express elevated levels of c-Fos after deprivation-induced REM sleep rebound (Sapin et al., 2009). As neurons labeled by c-Fos project to the vlPAG, this area may also promote REM sleep through inhibition of the vlPAG (Clément et al., 2012). Pharmacological suppression of the DPGi increased wakefulness and led to an activation of noradrenergic and adrenergic neurons including cells in the LC, indicating that DPGi GABAergic neurons inhibit adrenergic/noradrenergic neurons (Clément et al., 2014). Electrical stimulation of the nucleus prepositus hypoglossi (dorsal to the DPGi) causes an increase in REM sleep, which could be reversed by infusion of the GABA-receptor antagonist picrotoxin into the LC (Kaur et al., 2001), suggesting that inhibition of the LC by the dorsomedial medulla directly enhances REM sleep. Interestingly, calbindin-expressing neurons in the DPGi have recently been shown to be required for eye movements during REM sleep (Gutierrez Herrera et al., 2019).

The pons and midbrain also contain GABAergic REM sleep-active neurons. c-Fos immunohistochemistry, juxtacellular recordings, and calcium imaging demonstrated the existence of GABAergic REM-on neurons in the dorsolateral pons (Maloney et al., 1999; Lu et al., 2006; Boucetta et al., 2014; Cox et al., 2016), some of which project to the vlPAG/DpMe (Lu et al., 2006). Finally, GABAergic REM sleep-active neurons can also be found within the vlPAG/DpMe as shown by optrode recordings and c-Fos immunohistochemistry (Sapin et al., 2009; Weber et al., 2018). However, whether activation of this GABAergic subpopulation promotes REM sleep, possibly through local synaptic interactions with REM-off neurons, is still unknown. Altogether, inhibitory REM-on neurons have been discovered in multiple brain areas including medulla, pons, midbrain, and hypothalamus. A common mechanism by which these different areas promote REM sleep is inhibition of REM-off neurons in the vlPAG resulting in disinhibition of the SLD (Figure 2).

## Homeostatic Regulation of REM Sleep

### Behavioral Studies of REM Sleep Homeostasis

REM sleep is under homeostatic control as demonstrated in various mammalian species including mice, rats, cats, and humans (Siegel and Gordon, 1965; Beersma et al., 1990; Benington et al., 1994; Endo et al., 1997, 1998; Rechtschaffen et al., 1999; Franken, 2002; Shea et al., 2008). The buildup of a need for REM sleep in its absence can be demonstrated by selectively depriving animals of REM sleep, typically by awakening them at the first sign of REM sleep: The number of interventions required to stop the occurrence of REM sleep increases during the deprivation period (Endo et al., 1997, 1998; Shea et al., 2008). During the subsequent recovery sleep, the amount of REM sleep is increased above baseline levels to account for the amount lost during deprivation, unlike NREM sleep, which to a large extent is compensated for by an enhanced intensity of sleep, as reflected in increased EEG SWA (Borbély et al., 1981; Vyazovskiy et al., 2009). The homeostatic control of REM sleep is independent of the circadian clock: rats in which the suprachiasmatic nucleus (SCN) was lesioned still show a REM sleep rebound following total sleep or REM sleep deprivation (Mistlberger et al., 1983; Wurts and Edgar, 2000).

### Homeostatic Timing of REM Sleep

**FIGURE 3:**
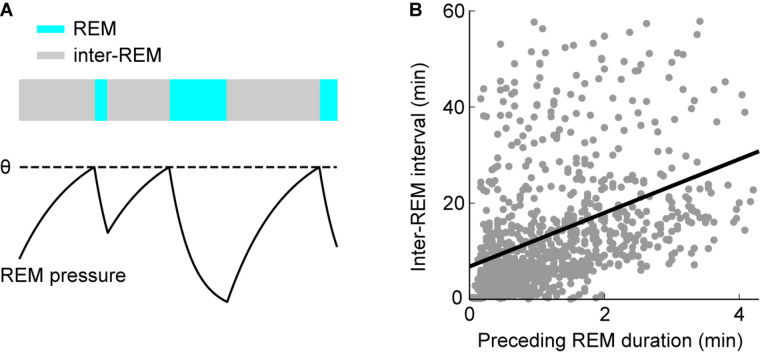
Homeostatic regulation of REM sleep timing. **(A)** Scheme illustrating the model for homeostatic regulation of REM sleep timing. Homeostatic REM sleep pressure accumulates in the absence of REM sleep during inter-REM periods and discharges during REM sleep. REM sleep is triggered once the REM sleep pressure reaches the threshold θ (dashed line). Consequently, longer REM periods precede longer inter-REM intervals. **(B)** Correlation between inter-REM interval and preceding REM sleep episode duration. Each point represents a single episode (*n* = 972 episodes from 27 mice). REM episodes and inter-REM intervals are positively correlated (linear regression: *R* = 0.39, *p* = 6.8 × 10^– 36^). The model in **(A)** may explain this positive correlation. **(B)** Reproduced from Weber et al. (2018).

In rats, 2 h or less of REM sleep deprivation are sufficient to trigger a rebound (Benington et al., 1994; Shea et al., 2008; Datta and Oliver, 2017). Therefore, the underlying homeostatic process that keeps track of the amount of REM sleep may also trigger REM sleep periods under natural conditions (Benington and Heller, 1994; Vivaldi et al., 1994). According to this view, a homeostatic propensity or pressure for REM sleep builds up in its absence, triggers a REM period if sufficient pressure has accumulated and then dissipates during REM sleep (Figure 3A). Consequently, it should take more time after a long REM period until the next REM sleep episode occurs: As more REM sleep pressure is dissipated during a longer episode, more time is required to accumulate sufficient pressure to re-enter REM sleep (Benington and Heller, 1994; Vivaldi et al., 1994). This predicted positive correlation between REM sleep duration and the following time until the next REM sleep episode (inter-REM interval) could indeed be verified in mice (Weber et al., 2018; Arthaud et al., 2020; Figure 3B), rats (Benington and Heller, 1994; Vivaldi et al., 1994, 2005; Gregory and Cabeza, 2002; Bassi et al., 2009), cats (Ursin, 1970), monkeys (Weitzman et al., 1965), and humans (Barbato and Wehr, 1998). More detailed analysis showed that the positive correlation is largely explained by the total duration of NREM sleep and not wakefulness during the subsequent inter-REM interval, indicating that, at least on a time scale of minutes in rodents, REM propensity accumulates during NREM sleep and not wakefulness (Benington and Heller, 1994; Vivaldi et al., 1994, 2005).

However, the notion that REM sleep pressure exclusively accumulates during NREM sleep contradicts other findings from extended sleep deprivation experiments in both rats (>12 h) (Rechtschaffen et al., 1999; Franken, 2002) and humans (>62 h) (Berger and Oswald, 1962; Kales et al., 1970). Deprivation of total sleep in these studies is followed by a rebound in REM sleep, suggesting that REM sleep pressure also builds up during wakefulness. The contrasting results on the role of wakefulness in REM sleep pressure regulation are reconciled in a model based on sleep deprivation in rats suggesting the existence of two homeostatic processes operating on different timescales (Franken, 2002): A short-term process, building up during NREM sleep and discharging during REM sleep, controls the timing of single REM sleep periods on a timescale of minutes. The daily amount of REM sleep is instead governed by a long-term process, which accumulates homeostatic pressure over a timescale of hours during both NREM sleep and wakefulness.

### Molecular Basis of REM Sleep Pressure

The physiological or molecular basis of the homeostatic regulation of REM sleep is still largely unknown. Recent studies suggest a prominent role of brain-derived neurotrophic factor (BDNF) in mediating the accumulation of REM sleep pressure. REM sleep deprivation for 3 h increased the expression of BDNF in the PPT and subcoeruleus nucleus (Datta et al., 2015). The levels of BDNF are positively correlated with the strength of the homeostatic drive, as quantified by the attempts to enter REM sleep during the deprivation phase (Datta et al., 2015; Barnes et al., 2017). Infusing inhibitors of BDNF-tropomyosin receptor kinase B (TrkB) receptors prevented an up-regulation of BDNF and suppressed the homeostatic rebound, suggesting that BDNF-TrkB signaling mediates the buildup of REM sleep pressure (Barnes et al., 2017). Moreover, REM sleep deprivation promoted phosphorylation of extracellular-signal-regulated kinase 1 and 2 (ERK1/2), which in turn resulted in increased BDNF expression (Datta and Oliver, 2017). These findings suggest that a positive molecular feedback loop might underlie the development of REM sleep pressure: The release of BDNF in the absence of REM sleep enhances BDNF-TrkB signaling, which triggers phosphorylation of ERK1/2, which in turn leads to increased expression of BDNF. It would be interesting to identify the neurons releasing and sensing BDNF and to unravel mechanisms causing a decrease of BDNF levels as REM sleep pressure discharges. Finally, an important question is whether this molecular process also operates on a timescale of minutes and might thus not only mediate the buildup of REM sleep pressure over longer time intervals, but also regulate the timing of REM sleep periods during natural sleep.

### Circadian Modulation of REM Sleep

Besides homeostatic mechanisms, the daily expression of REM sleep is also modulated by circadian influences. REM sleep propensity is largest toward the end of the rest phase, when sleep tendency is highest and coincides with the rising slope of the body temperature rhythm, which is also under circadian control (Czeisler et al., 1980; Dijk and Czeisler, 1995). REM sleep deprivation experiments in rats with SCN lesions suggest that the circadian clock facilitates REM sleep during the rest, but not the activity phase, as quantified by the number of attempts to enter REM sleep during deprivation (Wurts and Edgar, 2000). Lesioning orexin/hypocretin neurons led to an increase of REM sleep only during the dark period, suggesting that these neurons suppress REM sleep during the active phase (Kantor et al., 2009). Hence, the daily expression of REM sleep may depend on both facilitatory and suppressive mechanisms, respectively mediated by the SCN and orexin/hypocretin neurons.

## Quantitative Models of REM Sleep Regulation

### Reciprocal Interaction Model

**FIGURE 4:**
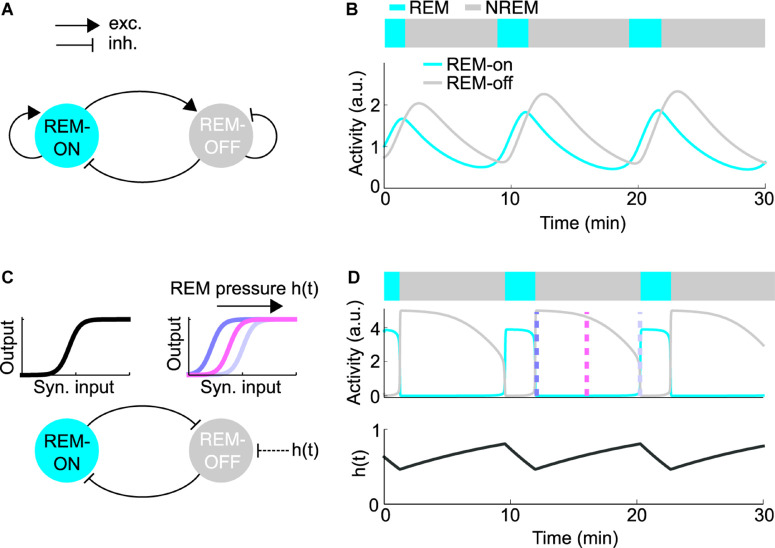
Quantitative models for REM sleep regulation. **(A)** Schematic of the reciprocal interaction (RI) model. Each node represents the average firing rate of the REM-on or REM-off population. The model was implemented as described in Diniz Behn et al. (2013). **(B)** Simulation of the RI model depicting the activity of the REM-on and REM-off population along with the resulting hypnogram. **(C)** Schematic of the mutual inhibition (MI) model. Inhibitory populations of REM-on and REM-off neurons mutually inhibit each other (bottom). The nodes represent the average firing rates of the REM-on and REM-off populations. Sigmoid input-output (i/o) functions describe the steady-state firing rate dependent on the inhibitory synaptic input (top). To exhibit oscillations, the model requires an oscillatory external input or parameter change. The accumulation of homeostatic REM sleep pressure, denoted as h(t), induces a right-wards shift of the REM-off i/o function throughout NREM sleep, resulting in reduced excitability of REM-off neurons. The model was implemented as described in Diniz Behn et al. (2013). **(D)** Simulation of the MI model. The average activity of the REM-on and REM-off population (middle) is depicted along with the resulting hypnogram (top) and the homeostatic REM sleep pressure h(t) (bottom), which determines the shape of the REM-off i/o function. The color-coded dashed lines represent the shape of the i/o functions as shown in **(C)**.

One of the first quantitative models describing the dynamics underlying the sleep cycle was proposed by McCarley and Hobson (1975). Their dynamical model reproduced the activity of two populations of neurons with an antagonistic firing pattern relative to REM sleep recorded in the pontine gigantocellular tegmental field (Hobson et al., 1975). In the original model, cholinergic neurons drive REM sleep as excitatory REM-on neurons, whereas monoaminergic neurons in the LC and DR suppress REM sleep as REM-off neurons (Figure 4A). The dynamics of the systems are described by Lotka-Volterra-type equations, which are also used to model predator/prey interactions. During NREM sleep, the monoaminergic neurons inhibit the cholinergic REM-on cells (Figure 4B). But, the inhibition of REM-on neurons slowly decays due to self-inhibition of the REM-off neurons, leading to disinhibition of the cholinergic neurons, resulting in a transition into REM sleep. The REM-on neurons, however, excite the REM-off neurons, ultimately leading to the termination of REM sleep.

### Mutual Inhibition Model

Based on subsequent studies pointing to more prominent roles of GABAergic REM-on and REM-off neurons in REM sleep control, recent models stress the importance of reciprocal inhibitory interactions between REM-on and REM-off neurons (Lu et al., 2006; Saper et al., 2010; Diniz Behn et al., 2013; Booth and Diniz Behn, 2014; Dunmyre et al., 2014; Weber, 2017; Héricé et al., 2019). The mutual inhibition (MI) or flip-flop model can be conceptualized as two populations of inhibitory neurons antagonizing each other (Figures 4C,D). According to the model, REM-off neurons are active during NREM sleep, while REM-on neurons are silent and vice versa during REM sleep. By switching back and forth between these two network states, the MI model is thought to generate repeated transitions between NREM and REM sleep. However, mutual inhibition between two populations alone is not sufficient to generate periodic alternations between two states (Diniz Behn et al., 2013). In its simplest analytical form, the REM-on and REM-off populations of the MI model are represented by their respective mean firing rates. The steady-state firing rate of each population is described by a non-linear, sigmoid input-output (i/o) function mapping the inhibitory synaptic input onto the mean firing rate (Figure 4C). Assuming fixed synaptic weights, the network will converge to a stable fixed point (corresponding to NREM or REM sleep), instead of generating an alternation between network states. Thus, to produce an alternation between NREM and REM sleep, the MI model requires an oscillatory external input or other parameter changes forcing the network to periodically transition between two different fixed points. A recent modeling study proposed that homeostatic REM sleep pressure, building up in the absence and discharging in the presence of REM sleep, acts as the external input that switches the MI network back and forth between NREM and REM sleep (Dunmyre et al., 2014). The REM sleep pressure is assumed to exert an inhibitory effect on the REM-off neurons by steadily reducing their excitability throughout NREM sleep (Figures 4C,D). Alternatively, oscillations can also be generated when REM sleep pressure excites the REM-on population. However, in the opposite configuration, where the REM-off population is inhibited by REM sleep pressure, the oscillatory behavior of the network was found to be more stable with respect to parameter changes and might thus be more robust (Diniz Behn et al., 2013).

### Anatomical Evidence for a Mutual Inhibition Network

Assuming that mutually inhibitory interactions control transitions into and out of REM sleep, a key question is which of the multiple REM-on and REM-off populations constitute the core MI network. Given the strong evidence for the necessary and sufficient role of vlPAG/DpMe GABAergic neurons in gating REM sleep (Petitjean et al., 1975; Sastre et al., 1996; Crochet et al., 2006; Lu et al., 2006; Vanini et al., 2007; Kaur et al., 2009; Sapin et al., 2009; Hayashi et al., 2015; Weber et al., 2015, 2018), these neurons may be identified with the REM-off node in the MI network. In addition, the vlPAG/DpMe is innervated by multiple inhibitory REM-on neurons in hypothalamus, pons, and medulla (Lu et al., 2006; Goutagny et al., 2008; Clément et al., 2012; Weber et al., 2015; Kroeger et al., 2019; Figure 2). In contrast, it is still unclear which of the inhibitory REM-on neurons are in turn inhibited by vlPAG/DpMe GABAergic neurons, thus completing the MI network. A previous study suggested that the vlPAG/DpMe forms a mutually inhibitory network with GABAergic REM sleep-active neurons in the dorsolateral pons (Lu et al., 2006). Knockdown of *Vgat* in this area, however, did not alter REM sleep (Krenzer et al., 2011). vlPAG neurons may directly inhibit REM-on neurons in hypothalamic areas or the ventral medulla through their axonal projections to these brain regions (Cameron et al., 1995a, b; Sirieix et al., 2012). Rabies virus-mediated retrograde tracing demonstrated that Lhx6 neurons in the ZI and MCH neurons in the LH are innervated by neurons in the ventral PAG (González et al., 2016; Liu et al., 2017). Whether these presynaptic neurons are GABAergic and suppress REM sleep through inhibition of the hypothalamic REM-on neurons, however, is still unknown. Alternatively, the connection from REM-off to REM-on neurons may be indirect. vlPAG GABAergic neurons innervate glutamatergic neurons in the SLD (Weber et al., 2018), which in turn project to the vM (Sirieix et al., 2012) and may thus excite inhibitory REM-on neurons in the ventral medulla (Figure 2).

### Modulation of REM-Off Activity by REM Sleep Pressure

**FIGURE 5:**
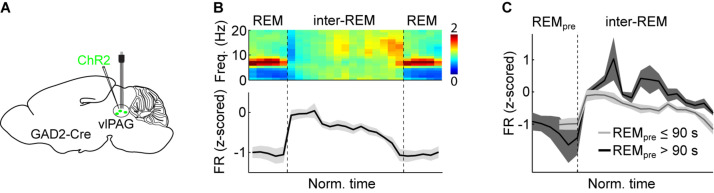
Experimental support for the MI model. **(A)** Scheme depicting electrophysiological recordings from vlPAG GABAergic neurons expressing ChR2 using an optrode (microelectrodes attached to an optic fiber). Viral vectors expressing Cre-dependent ChR2 were injected into the vlPAG of GAD2-Cre mice. Optrode recordings allow the experimenter to test whether a recorded unit is reliably driven by laser stimulation and thus can be classified as the ChR2-expressing cell-type. **(B)** Average normalized EEG spectrogram (top) and mean firing rate (z-scored) of REM-off vlPAG GABAergic neurons (bottom, *n* = 11) during two successive REM episodes and the inter-REM interval. Each REM sleep episode and inter-REM interval was temporally normalized to unit length. **(C)** Comparison of vlPAG activity during inter-REM interval following short (≤90 s) and long (>90 s) REM episodes. Following a long REM sleep period, the firing rate was significantly higher than following a short one. **(B,C)** Reproduced from Weber et al. (2018).

In the MI model, the alternation between NREM and REM sleep relies on the accumulation and discharge of homeostatic REM sleep pressure (Figure 4C). Assuming that REM sleep pressure lowers the excitability of REM-off neurons, their firing rates should be highest right after REM sleep (when REM sleep pressure is lowest), progressively decay during the course of the sleep cycle, and reach lowest activity levels right before entering the next REM period (when REM sleep pressure is highest; Figure 4D). Cell-type-specific recordings using optrodes and calcium imaging of GABAergic REM-off neurons in the vlPAG indeed revealed a slow, gradual decrease of their baseline activity during inter-REM intervals (Figures 5A,B), providing correlative evidence for the idea that homeostatic REM sleep pressure exerts an inhibitory effect on the activity of REM-off neurons (Weber et al., 2018).

Further analysis showed that the activity of vlPAG GABAergic neurons specifically decayed during NREM sleep and not during wakefulness within inter-REM intervals (Weber et al., 2018), consistent with the assumption that homeostatic REM sleep pressure specifically builds up during NREM sleep, and not wakefulness (Benington and Heller, 1994; Vivaldi et al., 1994). Moreover, chemogenetic experiments also indicate an involvement of GABAergic neurons in the vlPAG/DpMe in the homeostatic control of REM sleep. Chemogenetic activation of GABAergic neurons after 6 h of REM sleep deprivation blocked the rebound of REM sleep during recovery sleep, indicating that activating these neurons might erase the homeostatic need for REM sleep (Hayashi et al., 2015). Finally, in the brainstem of cats, REM sleep deprivation reduced the activity of presumable noradrenergic REM-off neurons in the LC, thus potentially facilitating transitions into REM sleep under conditions of increased REM sleep pressure (Mallick et al., 1990).

Assuming that the timing of REM sleep periods is under homeostatic control, more REM sleep pressure should be dissipated after a long REM period compared with a short one. Therefore, if REM-off neurons are indeed inhibited by REM sleep pressure, their activity should be higher the longer the preceding REM period. The firing rates of vlPAG REM-off neurons during the inter-REM interval were indeed higher following long REM episodes than after short ones (Figure 5C), and the mean firing rate during the inter-REM interval was significantly correlated with the preceding REM episode duration, providing further evidence for the notion that the activity of vlPAG GABAergic neurons reflects REM sleep pressure (Weber et al., 2018).

A further prediction by the MI network is that REM sleep periods can in principle be triggered via two parallel mechanisms: First, as long as accumulating REM sleep pressure progressively inhibits REM-off neurons, a NREM to REM transition will spontaneously occur without requiring the command from an additional master node. Alternatively, any external inhibitory/excitatory input to the REM-off/REM-on populations of the MI network can also trigger REM sleep at any time if it sufficiently inhibits/excites the REM-off/REM-on population. Although not necessary for the generation of REM sleep, such external inputs may nevertheless strongly modulate its timing and duration. External inputs to the core REM network may serve to optimally adjust the amount of REM sleep to needs arising from environmental factors such as the ambient temperature (Komagata et al., 2019) and from internal processes such as learning or emotional coping (Sanford et al., 2010; Datta and O’Malley, 2013).

## Discussion

The MI model posits that REM sleep pressure acts on the excitability of REM-off neurons and thereby times the onset of REM sleep periods. Behavioral data analysis and recent *in vivo* recordings of REM-off neurons in the vlPAG provided correlational evidence for the homeostatic regulation of REM sleep timing (Benington and Heller, 1994; Vivaldi et al., 1994; Weber et al., 2018). However, experiments to further validate this model are hampered by the fact that no easily measurable marker for REM sleep pressure has been discovered to date. Further complicating the situation, comparing total sleep with REM sleep deprivation experiments suggests the existence of two homeostatic processes operating on different timescales (Franken, 2002). It is still unclear whether the mechanisms regulating long-term REM sleep pressure, which sets the daily amount of REM sleep, differ from or overlap with the homeostatic mechanisms controlling the timing of REM sleep periods. Identifying a marker for REM sleep pressure would enable researchers to delineate the mechanisms underlying the buildup of REM sleep pressure and to directly test its causal contribution to REM sleep timing. Furthermore, carefully studying the *in vivo* dynamics of REM-on and REM-off neurons and how their manipulation affects the statistics of the sleep architecture will help to further verify or refute the predictions of physiologically based models.

Numerous studies consistently point to the importance of the vlPAG as a major REM-off center (Petitjean et al., 1975; Sastre et al., 1996; Crochet et al., 2006; Vanini et al., 2007; Sapin et al., 2009; Hayashi et al., 2015; Weber et al., 2018), whereas various REM-on populations have been identified throughout the hypothalamus and brainstem (Jego et al., 2013; Konadhode et al., 2013; Tsunematsu et al., 2014; Weber et al., 2015; Kroeger et al., 2019). Further work is necessary to identify which of the REM-on populations are part of an MI network or which ones only unidirectionally interact with REM-off neurons. A prediction by the MI model is that the key REM-on neurons, which are an integral part of the REM circuitry, should be under inhibitory control by the REM-off population.

As the duration of REM sleep influences the amount of subsequent NREM sleep, delineating the mechanisms underlying the maintenance of REM sleep is important to understand the regulation of the sleep cycle. While activation of various REM-on populations including neurons in dorsal and ventral medulla and hypothalamic MCH neurons (Kaur et al., 2001; Jego et al., 2013; Weber et al., 2015) have been shown to increase the duration of single REM sleep episodes, the mechanisms maintaining REM sleep, or conversely the circuits responsible for its termination, are not well understood. Particularly in rodents, the duration of REM sleep and the sleep cycle is highly variable (Trachsel et al., 1991). Determining the neural interactions that maintain and terminate REM sleep will therefore provide important insights into how changes in the underlying circuits may give rise to the large variability of mammalian sleep within the same and across species.

While statistical analysis of sleep data favors models in which REM sleep and consequently the sleep cycle is driven by a homeostatic process, the existence of a neural oscillator cannot be excluded. Particularly, the strikingly regular oscillation between two distinct brain states, reminiscent of NREM and REM sleep in a reptile, the Australian dragon *Pogona vitticeps* (Shein-Idelson et al., 2016), rather seems to suggest the existence of an ultradian oscillator circuit in the vertebrate brain. Besides the ultradian rhythm, regular infraslow rhythms in the minute range have been described in humans and rodents (Lecci et al., 2017; Watson, 2018). Both rodents and humans show a pronounced 0.02 Hz oscillation in the sigma power of the EEG. In addition, a 20–40 s rhythm (cyclic alternating pattern) has been described in the human EEG occurring during NREM sleep (Terzano et al., 1985), and hemodynamics in resting state networks also shows a distinct ultradian rhythm (Fox and Raichle, 2007; Ma et al., 2016). It would be interesting to know to what extent these rhythms are synchronized with the sleep cycle or play a role in regulating the timing of REM sleep. Besides these slow ultradian rhythms, it is also important for future studies to understand how the circuits and homeostatic processes timing the recurrence of REM sleep interact with networks regulating fast oscillations on a millisecond-to-second timescale, such as slow waves, sleep spindles, or hippocampal theta oscillations. Recent studies demonstrated prominent roles of the supramammillary nucleus and the medial septum in the control of theta oscillations during REM sleep (Boyce et al., 2016; Billwiller et al., 2019). How neurons in these areas are connected to the REM control circuits or are possibly influenced by homeostatic processes awaits future research.

We believe that for the future, a model-guided approach that builds on existing knowledge to constrain conceptual models and tests their predictions experimentally will help to determine the core circuits and homeostatic mechanisms controlling REM sleep and the temporal dynamics giving rise to the ultradian sleep cycle. Understanding how external circuits and molecular factors influence the core network and how these circuits are in turn modulated by homeostatic needs for REM sleep will provide crucial insights into the functional roles of REM sleep.

## Author Contributions

FW and S-HP wrote the manuscript and made the figures. Both authors contributed to the article and approved the submitted version.

## Conflict of Interest

The authors declare that the research was conducted in the absence of any commercial or financial relationships that could be construed as a potential conflict of interest.
